# Gelatin-Based Hydrogels for Organ 3D Bioprinting

**DOI:** 10.3390/polym9090401

**Published:** 2017-08-30

**Authors:** Xiaohong Wang, Qiang Ao, Xiaohong Tian, Jun Fan, Hao Tong, Weijian Hou, Shuling Bai

**Affiliations:** 1 Department of Tissue Engineering, Center of 3D Printing & Organ Manufacturing, School of Fundamental Sciences, China Medical University (CMU), No. 77 Puhe Road, Shenyang North New Area, Shenyang 110122, China; aoqiang00@163.com (Q.A.); xhtian@cmu.edu.cn (X.T.); jfan@cmu.edu.cn (J.F.); tongh007@cmu.edu.cn (H.T.); wjhou@cmu.edu.cn (W.H.); baishuling@cmu.edu.cn (S.B.); 2 Center of Organ Manufacturing, Department of Mechanical Engineering, Tsinghua University, Beijing 100084, China

**Keywords:** 3D bioprinting, gelatin-based hydrogels, rapid prototyping (RP), organ manufacturing, implantable bioartificial organs

## Abstract

Three-dimensional (3D) bioprinting is a family of enabling technologies that can be used to manufacture human organs with predefined hierarchical structures, material constituents and physiological functions. The main objective of these technologies is to produce high-throughput and/or customized organ substitutes (or bioartificial organs) with heterogeneous cell types or stem cells along with other biomaterials that are able to repair, replace or restore the defect/failure counterparts. Gelatin-based hydrogels, such as gelatin/fibrinogen, gelatin/hyaluronan and gelatin/alginate/fibrinogen, have unique features in organ 3D bioprinting technologies. This article is an overview of the intrinsic/extrinsic properties of the gelatin-based hydrogels in organ 3D bioprinting areas with advanced technologies, theories and principles. The state of the art of the physical/chemical crosslinking methods of the gelatin-based hydrogels being used to overcome the weak mechanical properties is highlighted. A multicellular model made from adipose-derived stem cell proliferation and differentiation in the predefined 3D constructs is emphasized. Multi-nozzle extrusion-based organ 3D bioprinting technologies have the distinguished potential to eventually manufacture implantable bioartificial organs for purposes such as customized organ restoration, high-throughput drug screening and metabolic syndrome model establishment.

## 1. Introduction

In the human body, an organ is a collection of tissues that unite as a structural entity to serve one or several common functions [1]. Ordinarily, an organ is composed of more than three tissues, i.e., the main (or parenchymal) tissue as well as stromal, and sporadic tissues, in which the main tissue is specific for unique functions. For example, in the heart, the main tissue is myocardium with blood-pumping function, while the sporadic tissues include the blood vessels, nerves, and connective tissues which help the myocardium to perform the blood-pumping function [2]. At present, the only effective therapy for an organ defect/failure is through allograft organ transplantation.

Allograft organ transplantation is a medical procedure in which an organ is removed from one individual to another (i.e., the recipient or patient), to replace a damaged/missing (i.e., defect/failure) organ [3]. This is one of the major breakthroughs in modern surgery. Through transplant operations, numerous patients have regained their lives with the transplanted organs. Nevertheless, the success rate in some vital organ transplants, such as the heart, lung and liver, turn out to be far from satisfactory [4]. One of the greatest problems in these organ transplants is the acute or chronic rejection, which means that the body tends to fight off, or reject, the organs from another body [5]. Over the last several decades, numerous scientists throughout the world have explored various technologies to manufacture bioartificial organs to repair/restore the defect/failure organs [6,7,8,9,10].

Three-dimensional (3D) bioprinting is the process of creating cell patterns in a confined space using 3D printing technologies, where cell function and viability are preserved within the printed construct [11,12]. The major advantage of 3D bioprinting technologies in “organ manufacturing” is to automatically produce bioartificial organs using heterogeneous cell types or stem cells along with other biomaterials to repair, replace or regenerate the defect/failure organs [13,14,15,16].

Gelatin is a water-soluble protein derived from natural polymer collagen. Gelatin-based hydrogels, such as gelatin, gelatin/alginate, gelatin/chitosan, gelatin/hyaluronan, gelatin/fibrinogen, gelatin/alginate/fibrinogen, and gelatin/alginate/fibrinogen/hyaluronan, have unique features, such as excellent biocompatibilities, rapid biodegradabilities and nonimmunogenicities, in clinical applications. These hydrogels have acted as the extracellular matrices (ECMs) in organ 3D bioprinting technologies and played a critical role in various pre-defined physical (i.e., structural and morphological), chemical, and biological functionality realization [17,18,19,20,21,22,23,24].

In this review, the intrinsic/extrinsic properties of the gelatin-based hydrogels in organ 3D bioprinting technologies are outlined [25,26,27,28,29,30,31,32,33,34,35]. The state of the art of the physical and chemical crosslinking techniques to improve the mechanical properties of the gelatin-based hydrogels are highlighted. Among all the 3D bioprinting technologies, multi-nozzle extrusion-based 3D bioprinting has become a powerful tool for manufacturing large scale-up vascularized organs with hierarchical internal/external structures, gradient material constituents (or components) and multiple physiological functions, which have the potential to be widely used in many biomedical areas, such as, the scale-up and customized organ regenerative medicine, high throughput drug screening and energy metabolic syndrome analysis [36,37,38,39,40].

## 2. Properties of the Gelatin-Based Hydrogels

### 2.1. Origin of Gelatin

**Figure 1:**
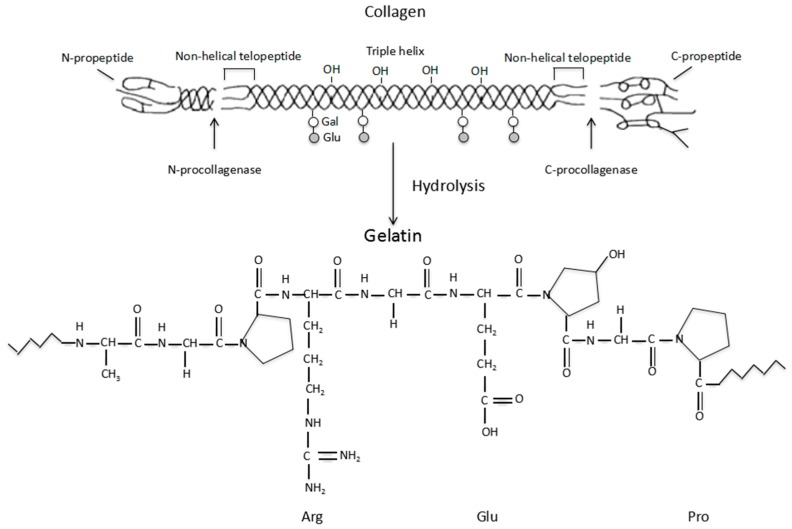
Schematic description of the collagen hydrolysis to gelatin.

Gelatin is a biodegradable polypeptide derived from the partial hydrolysis of collagen, which is a fibrous protein located mainly in the connective tissues within the body [41]. A schematic illustration of the collagen hydrolysis to gelatin is shown in Figure 1. An important function of collagen is to maintain the integrity of the connective tissues, such as the bones, cartilage, corneas, tendons, ligaments, blood vessels and dentin. There are at least 16 different collagen types in the human body. The most prominent types are the Type I, II and III (or collagen I, II and III), which make up approximately 80 to 90% of all the collagens in the body. Typically, type I collagen consists of three spiral polypeptide chains, about 30 nm in length, and 1.5 nm in diameter [42]. The typical triple helix structure of the type I collagen is composed of two α-chains and a β-chain [43,44]. Additionally, collagen has low antigenicity (i.e., immunogenicity), which arises from the determinant polypeptide structures in the three spiral chains as well as the central areas of the molecules. This has greatly limited its application in biomedical fields.

### 2.2. Properties of Gelatin

Several types of gelatin exist with different compositions depending on the source of the collagen and the hydrolytic treatment methods. For example, mammalian derived gelatins from pig and bovine have been widely exploited in regenerative medicine over the last several decades [45]. These gelatins have similar polypeptide structures with human beings. Meanwhile, fish derived gelatins have a significantly lower melting point, lower gelling temperature, lower thermal stability and higher viscosity. These gelatins, therefore, have a relatively lower content of peptide repetitions (or amino acid residues), such as prolinamide and hydroxyprolinamide, in their polypeptide chains.

The hydrolytic processes of collagen I can be classified into three groups: physical, chemical and enzymatic. The entire procedure from collagen I to gelatin can be defined into three stages (or phases): pretreatment of the raw material, extraction of the gelatin and purification and/or drying of the product [46]. Depending on the protocols used for collagen I pretreatment prior to the extraction process, two main types of gelatin can be produced: Type A and Type B. Type A gelatin, with an isoelectric point of 9.0, is derived from the acidic hydrolysis of collagen I, using sulfuric or hydrochloric acid. Type B gelatin, with an isoelectric point of 5.0, is derived from the alkaline hydrolysis of collagen I, using alkaline liquid (NaOH). In these cases, asparagine and glutamine amide groups in the collagen I molecules are hydrolyzed into carboxyl groups and resulted in aspartate and glutamate residues in the gelatin molecules [47].

During the hydrolytic processes, the typical triple helix structure of the collagen I is partly broken into single-stranded polymeric molecules (single chains). The average molecular weight of gelatin is between 15,000 and 400,000 Daltons [45]. Theoretically, all gelatins, no matter where they come from, are composed of Glycine-X-Y peptide triplets repetitions, where, X and Y can be any amino acid, but proline for X- and hydroxyproline for Y- positions are the most common formulations. The amino acid composition and sequence in the single chains can vary depending largely on the origin of the gelatin, and this in turn influences its final properties [46].

Thus, gelatin is a mixture of polypeptides, in which about 20 amino acids are connected by peptide bonds. The average length and molecular weight of the polypeptide gelatins depend on several factors, such as the origins of the raw materials, the pretreatment methods and the hydrolytic processing parameters (e.g., the pH, temperature and time) [45]. There are strong non-covalent interactions, such as van der Waal forces, hydrogen bonds, and electrostatic and hydrophobic interactions among the individual gelatin chains. Compared to its progenitor collagen I, the immunogenicity of gelatin is much lower due to the damage of the triple helix structures as well as the degradation of the propeptides [48].

### 2.3. Properties of Gelatin-Based Hydrogels

Unlike its progenitor collagen I, which is weakly acid soluble, gelatin is a water soluble natural polymer that can absorb 5–10 times the equivalent weight of water. When the temperature is increased, the dissolution speed can be accelerated to certain degree. Gelatin solution has an amphoteric behavior because of the presence of alkaline amino acids and acid functional groups. The electrostatic charge of gelatin solution varies depending on the treatment protocols used for gelatin extraction, which gives the gelatin solution different isoelectric points [49].

The behavior of a gelatin solution depends on several factors, such as temperature, pH, concentration and preparation method. A typical property of the gelatin solution is the capability to be gelled at low temperature (about 20–30 °C) by cooling to form hydrogels. This is a sol-gel transition process, which refers to gelation. During the gelation process, locally ordered regions among the gelatin molecules take place, that are subsequently joined by non-specific bonds, such as hydrogen, electrostatic and hydrophobic bonds. The resultant hydrogel has a unique thermo-reversible (i.e., thermally responsive or thermo-sensitive) character, since the non-specific bonds can be easily broken by heating. The unique property of the gelatin solution provides the gelatin-based hydrogels with a unique property, i.e., to be printed and stacked (overlapped or piled up) based on the computer aided design (CAD) model in a controlled manner [17,18,19,20,21,22].

## 3. 3D Bioprinting Technologies

### 3.1. Introduction of Rapid Prototyping (RP)

First developed in the 1980s, RP technology advanced using several methods that apply CAD files to create 3D objects [50]. These methods are unique in their layer-by-layer adding and bonding material fashions to form solid 3D objects [51]. Through the history, there are many different names for RP technologies, such as the additive manufacturing (AM), solid freeform fabrication (SFF), increasing material manufacturing (IMM), stereolithography (SLA) and layered manufacturing (LM). Recently, these have been substituted by 3D printing or 3D bioprinting in some research areas, such as, the medical engineering and tissue engineering [52,53,54]. Particularly, 3D bioprinting technology prints living cells together with other biomaterials, such as polymers and growth factors, under the instructions of CAD models. However, the term RP is still popular in the industry for digital manufacturing procedures. No matter in what format it appears, the basic principle of the RP technologies is to assemble materials in layers under the instruction of CAD files [55,56,57,58,59,60].

### 3.2. Classification of 3D Bioprinting Technologies

According to the classification of RP technologies, 3D bioprinting technologies can been divided into three main classes based on the working principles: extrusion-based 3D bioprinting, inkjet-based 3D bioprinting and laser-assisted based 3D bioprinting. The main objective of these 3D bioprinting technologies is to print living cells in layers using CAD models to produce bioactive constructs. Recent advances in the development of the 3D bioprinters have significantly enhanced their applications in producing scale-up tissues and organs, such as the skin, myocardium, nerve, liver, cartilage, bone, and blood vessel [61,62,63]. Each of the 3D bioprinting technologies has its own intrinsic advantages and disadvantages in organ 3D bioprinting with respect to the automaticity, printability, scalability, accuracy, and availability of ideal “bioinks” [64,65,66,67,68].

### 3.3. Advanced Organ 3D Bioprinting Technologies

**Figure 2:**
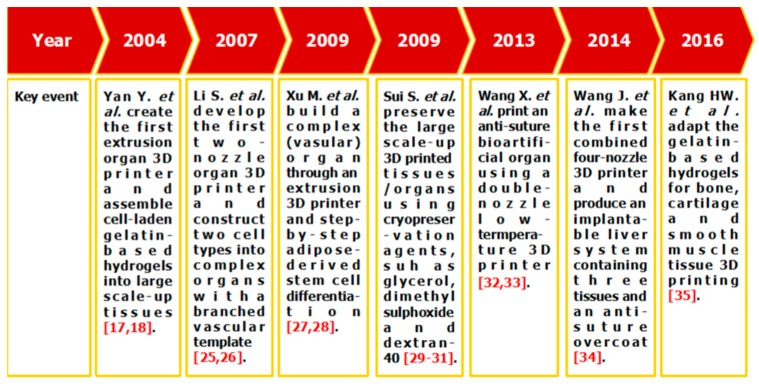
Histoical events of organ 3D bioprinting technologies.

**Figure 3:**
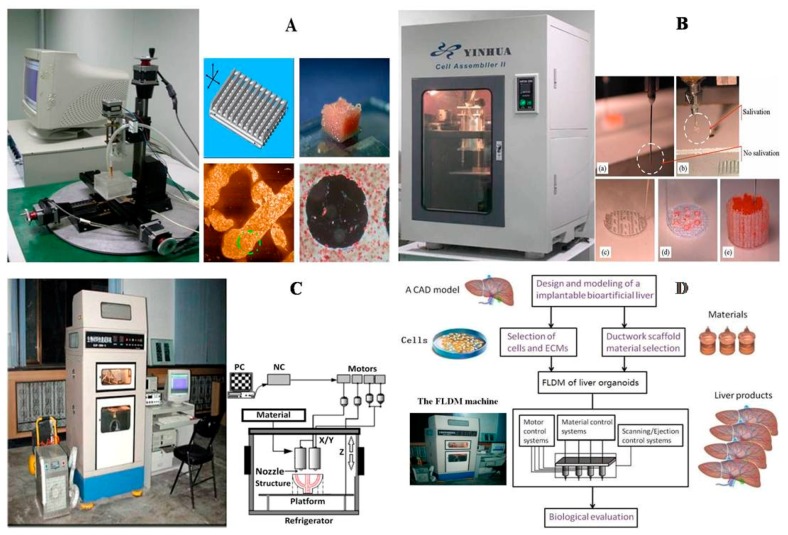
A schematic description of several pioneered 3D bioprinters made in Tsinghua Unversity, Prof. Wang’s laboratory: (**A**) hepatocytes and/or adipose-derived stem cells (ADSCs) in the gelatin-based hydrogels were first printed into large scale-up tissues in 2004 using the single-nozzle 3D bioprinter [17,18,19]; (**B**) two cell types in the gelatin-based hydrogels were printed simultaneously into large scale-up organs in 2007 [24]; (**C**) both cell containing natural gelatin-based hydrogel and synthetic polymer systems were printed into large scale-up vascularized organs with a branched vascular template, that can be sutured to the host vasculatures, using the home-made double-nozzle low-temperature deposition manufacturing (DLDM) system (i.e., DLDM 3D bioprinter). An elliptical hybrid hierarchical polyurethane and cell/hydrogel construct was first produced using the DLDM 3D bioprinter [30,31]; (**D**) a schematic description of the modeling and manufacturing processes of four liver constructs with a four-nozzle low-temperature 3D bioprinter [15].

Among all the available 3D bioprinting technologies, extrusion-based 3D bioprinting technology has been advanced in various organ manufacturing areas due to the wide integrated, inestimable sophisticated and extensive automated properties [13,14,15]. Some of the multi-nozzle extrusion-based 3D bioprinting technologies have unique and tremendous capabilities in many biomedical applications, such as, defect/failure organ restoration, pathological organ modeling, disease organ diagnoses, and drug efficacy tests (Figure 2 and Figure 3) [13,14,15,69,70,71,72].

In 2004, for the first time, cells encapsulated in the biodegradable gelatin-based hydrogels were printed to large scale-up grid 3D structures with nutrient and metabolite exchanging channels (Figure 3A) [17,18]. Subsequently, a protocol for organ 3D bioprinting with multiple steps of polymer crosslinking and cocktail stem cell engagement in a predefined construct was established [27,28]. Adipose-derived stem cells (ADSCs) in the grid 3D printed construct were first induced into both endothelial and adipose tissues with the spatial effects. It is therefore the first 3D bioprinted artificial organ with a confluent endothelial cell layer on the surface of the go-through channels which could not be realized through other technologies at that period. This is no doubt a long historical dream of human beings to manufacture large vascularized tissues and/or organs.

In 2007, it was the first time that a bioartificial organ (i.e., prevascularized hepatic tissue) was printed using a two-nozzle extrusion-based 3D bioprinter [25,26]. It was also the first report that multiple cell types, such as ADSCs and hepatocytes, were assembled into large vascularized liver tissues with a uniaxial branched vascular system. In 2009, it was the first time that 3D bioprinting technologies were combined with cell cryopreservation techniques successfully to store and preserve the large scale-up bioartificial tissues and/or organs [29,30,31]. This can greatly save the manual labour, material resources and financial expenses in human organ manufacturing areas. In 2013, it was the first research that natural cell-laden gelatin-based hydrogel and synthetic polymer systems were printed into hierarchical constructs with a predesigned vascular template for in vivo implantation [32,33]. With the incorporation of synthetic polymers, large scale-up implantable organ manufacture comes true with anti-suture and biostable capabilities. Later in 2014, it was the first time that a more complicated organ with more than three cell types and/or material systems in a predefined construct was developed using a combined multi-nozzle 3D bioprinter [34]. This is a long-awaited breakthrough in biomedical fields.

**Figure 4:**
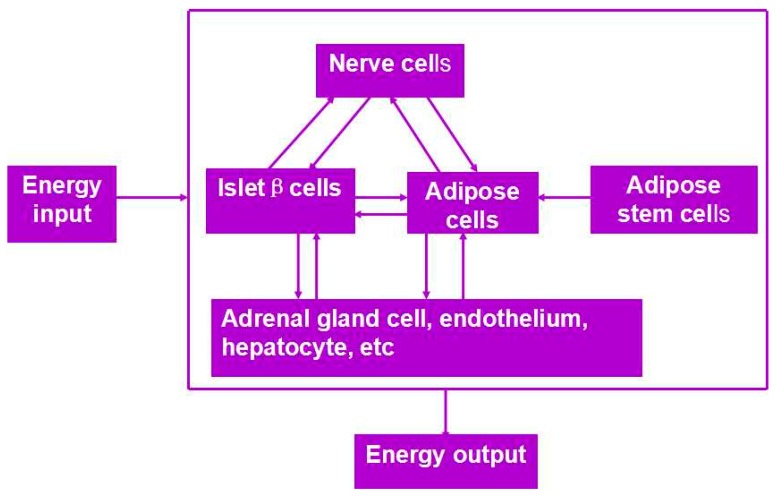
An energy metabolism model established through the adipose-derived stem cell (ADSC) laden gelatin-based hydorgel double-nozzle 3D bioprinting technology.

**Figure 5:**
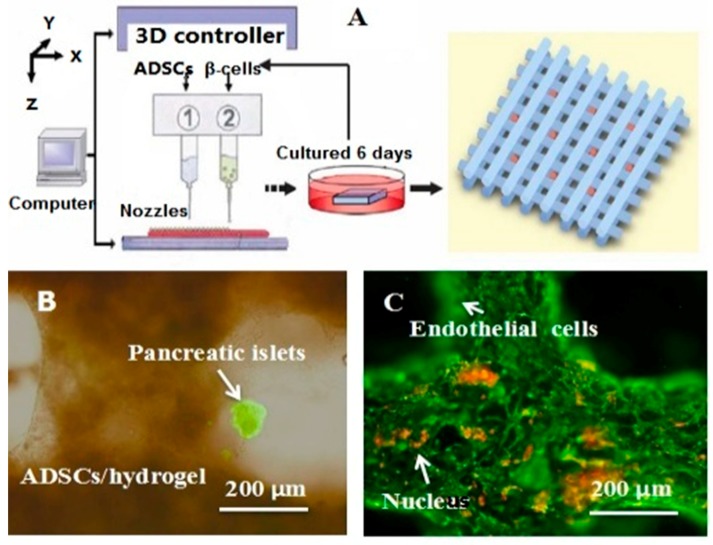

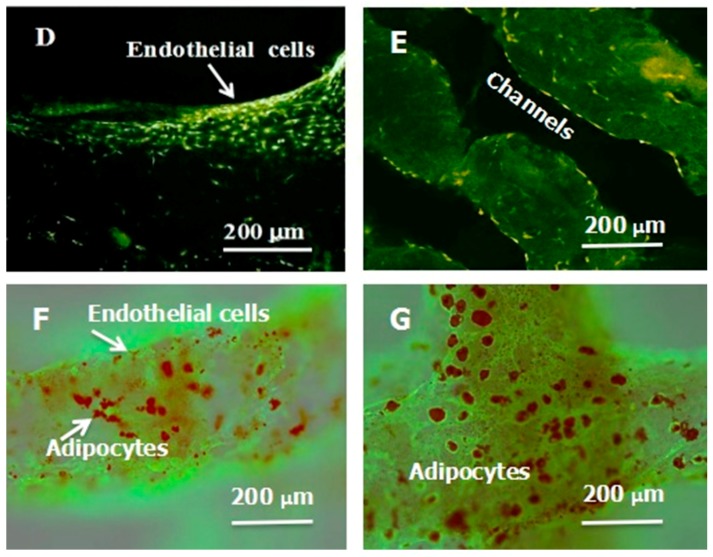
A large scale-up vascularized organ (i.e., vascularized adipose tissue incorporation with pancreatic islets) constructed through the double-nozzle gelatin-based hydrogel organ 3D bioprinting technology [27,28]: (**A**) the construction processes of the large scale-up vascularized organ (i.e., multicellular organ), based on the adipose derived stem cell (ADSC) laden gelatin/alginate/fibrin hydrogel 3D bioprinting and the subsequent β-cells (in the pancreatic islets) seeding procedures; (**B**) a multicellular construct, containing both ADSCs encapsulated in the gelatin/alginate/fibrin hydrogel before epidermal growth factor (EGF) engagement and pancreatic islet seeding in the predefined channels (immunostaining with anti-insulin in green); (**C**) immunostaining of the 3D construct with mAbs for CD31+ cells (i.e., mature endothelial cells from the ADSC differentiation after 3 days culture with EGF added in the culture medium) in green and pyrindine (PI) for cell nuclei (nucleus) in red; (**D**) immunostaining of the 3D construct with mAbs for CD31+ cells (i.e., mature endothelial cells) in green, having a fully confluent layer of endothelial cells (i.e., endothelium) on the surface of the predefined channels; (**E**) a vertical image of the 3D construct showing the fully confluent endothelium (i.e., endothelial cells) and the predefined go-through channels; (**F**) immunostaining of the 3D construct with mAbs for CD31+ cells in green and Oil red O staining for adipocytes in red, showing both the heterogeneous tissues coming from the ADSC differentiation after a cocktail growth factor engagement (i.e., on the surface of the channels the endothelium coming from the ADSCs differentiation after being treated with EGF for 3 days, deep inside the gelatin/alginate/fibrin hydrogel the adipose tissue coming from the ADSCs differentiation after being subsequently treated with insulin, dexamethasone and isobutylmethylxanthine (IBMX) for another 3 days. Spatial effect is prominent for the 3D printed constructs); (**G**) a control of (**F**), showing all the ADSCs in the 3D construct differentiated into target adipose tissue after 3 days treatment with insulin, dexamethasone and IBMX, but no EGF.

Besides organ manufacturing, these extrusion-based 3D bioprinting technologies provide additional high-tech platforms for other biomedical purposes. For example, drug screening has experienced a long history. One of the major obstacles in drug discovery for metabolic syndrome (MS) is the lack of in vitro models that capture the complex features of the disease. This was the first time, an energy metabolism model was established using an extrusion-based 3D bioprinting technology (Figure 4 and Figure 5) [27,28]. ADSCs encapsulated in the gelatin-based (i.e., gelatin/alginate/fibrinogen) hydrogels were printed and induced to differentiate into adipocytes and endothelial cells in a predefined 3D construct. Pancreatic islets were then deposited at the designated places in the 3D construct to mimic the adipoinsular axis with the induced adipocytes. The results indicated that drugs known to have effects on MS were shown to have an accordant effect in this model. Compared with traditional two-dimensional cell cultures, cell states in the 3D construct were much more similar to those in the native human organs. Many more physiological and pathophysiological features of energy metabolism as well as MS in vivo could be captured by the multicellular 3D construct. It is also the first large scale-up 3D printed vascularized bioartificial organ in history with a fully confluent endoderm on the surface of the predefined channels.

Back to 2003, the first center of organ manufacturing was set up in Tsinghua University by Professor Wang, the founder of “organ manufacturing”, with the purpose to produce bioartificial organs using advanced building technologies. The term “organ manufacturing” can be defined in a broad sense as the generation of organ substitutes using available biomaterials either with or without cells. “Organ manufacturing” can also be defined in a narrow sense, that is to produce bioartificial organs with living cells, along with other biomaterials. A prominent character of “organ manufacturing” is that the physical and/or chemical properties of the starting materials change during and/or after the construction processes. While those processes without any changes in the physical and/or chemical properties of the starting materials can only be called fabrication, processing or machining [13,14,15,64,65,66,67,68,73,74,75,76,77].

Over the last decade, two distinguished “organ manufacturing” strategies have been extensively explored by Prof. Wang in Tsinghua Univeristy [13,14,15,64,65,66,67,68,73,74,75,76,77]. These strategies include: (1) the combined multi-nozzle 3D bioprinting technologies [64,65,66,67,68]; and (2) the orderly addition of combined mold systems [73,74,75,76,77]. Either of the strategies can potentially bring about or has brought about a great revolution in biomedical fields with regard to improving the quality of life and extending the average life span (or expectancy) of human beings.

Consequently, a great deal of pioneering work has been conducted by Prof. Wang and her students. More than 120 articles have been published, with respect to the two series of new technologies, theories and principles in complex organ manufacturing, such as (1) “organ manufacturing” like building a nuclear power station, advanced construction tools, such as the combined multi-nozzle 3D bioprinting technologies and the orderly addition of combined mold systems are the key factors [13,14,15,64,65,66,67,68,73,74,75,76,77]; (2) a functional vasculature, including arteries, capillaries, and veins, with a confluent endoderm can be constructed using the stem cell engagement techniques [13,14,15]; (3) multiple physiological functions of a complex organ, such as, the liver, heart and kidney, can be achieved using both natural and synthetic polymers [13,14,15,64,65,66,67,68,69,70,71,72,73,74,75,76,77].

For instance, some arbitrary bioartificial organs with highly organized geometries, tailored “bioinks”, and main physiological functions have been constructed automatically and interactively. These technologies are especially useful in large scale-up and customized organ 3D bioprinting, in which CAD models established from expert experiences, or patient-specific data, including micro computed tomography (µCT), magnetic resonance imaging (MRI) and X-ray information, can be employed to guide the 3D bioprinting procedures [39,78,79,80,81,82].

## 4. Principles for Organ 3D Bioprinting

### 4.1. Basic Requirements for Organ 3D Bioprinting

As mentioned above, different 3D bioprinting technologies have different requirements for organ manufacturing. The fundamental issues for a typical organ 3D bioprinting is to recapitulate the essential morphological, physical (mainly mechanical and environmental), chemical, and biological properties of a native organ (i.e., its counterpart). To produce an organ substitute with a whole spectrum of physiological functions, several basic issues must be addressed first: (1) a powerful construction tool, such as the multi-nozzle 3D printer and orderly addition of combined mold system, is necessary to assemble multiple biomaterials, including diverse cell types, growth factors, and other bioactive agents, into a predefined 3D structure; (2) a functional soft-/hard-ware is preferential to automatically accomplish the intricate geometrical points (or patterns) of the target organ, including the vascular and nerve networks; (3) a large enough source (amount) of cells, especially multipotential stem cells as well as pertinent growth factors; (4) implantable natural and synthetic polymers, such as the gelatin, polyurethane (PU) and poly(lactic acid-*co*-glycolic acid) (PLGA), with excellent biocompatibilities to support multiple cellular activities, diverse tissue formation, multiple tissue coordination, and anti-suture anastomoses; (5) the ability to grasp the main characters of the target organs, with respect to the cellular environments, architectural structures and biological functions. Among all the basic issues, a powerful 3D printer, printable polymeric “bioinks” and enough cell sources are the key elements for a fully automated organ manufacturing process [13,14,15,64,65,66,67,68,73,74,75,76,77].

### 4.2. Organ 3D Bioprinting Procedures

A successful organ 3D bioprinting procedure requires several steps to implement: (1) structural blueprint predesigning; (2) starting material and construction tool preparation, including a capable multi-nozzle 3D printer and polymerous “bioinks” (including cells, polymers and growth factors); (3) 3D bioprinting; (4) post-printing organ maturation [13,14,15,64,65,66,67,68,73,74,75,76,77].

### 4.3. Blueprint Design

In the human body, each organ has its own anatomical structures, tissue types and physiological functions. Each organ needs to be particularly designed with special cell types, polymeric components, growth factors, and hierarchical architectures. To our knowledge, the main task of blueprint designing is to grasp the main characters of the target organ, but not all the minor details. For example, when we consider the liver construction, the most indispensable parts are the large vascular and bile duct networks that cannot be omitted in a liver lobe. Meanwhile, the detailed structures of capillary network can be neglected. In another words, every particular lobular architecture, does not need to be mimicked in detail. The capillary network can form spontaneously by self-assembling suitable cells/hydrogels under specific conditions if there is enough space. Thus, the incorporation of the bile duct network in the construct is much more important than the elaborate arrangement of every cell or each cell type in a particular lobular. This means that the macroscale properties of an organ are more important than the microscale features [13,14,15,64,65,66,67,68,73,74,75,76,77].

In general, the most indispensable parts (that cannot be omitted) for a vascularized organ, such as the liver, kidney and stomach, are the parenchymal tissues and the vascular networks. Tuned growth and remodeling frameworks of different tissues should be treated seriously. The coordination design for heterogeneous tissues in a 3D construct is extremely important for the full functionality realization [13,14,15,64,65,66,67,68,73,74,75,76,77]. Sometimes, a nervous network is also needed to be incorporated to support the vascular network as well as other tissues [32]. The CAD models containing a structured tree of information have played important roles for each organ blueprint designing. During the blueprint designing stage, physical (including morphological and architectural), chemical, and biological information of an organ may be collected from the clinical µCT, MRI or X-ray data. This may also come from expert experience. The digital data of a 3D image need to be simplified and translated into an actual physical entity. This CAD model can be used to guide the 3D bioprinting process. For a more complex organ, such as the brain, heart and skin, the vascular and nervous networks all need to be particularly designed with tuned growth and remodeling frameworks [13,14,15,64,65,66,67,68,73,74,75,76,77].

### 4.4. Mutinozzle 3D Printer and Polymerous “Bioink” Preparation

Each individual organ in the human body has its own requirements for 3D bioprinting. Every organ 3D bioprinter has its own prerequisites with respect to the viscosity, gelation kinetics and rheological properties of the employed “bioinks”. A powerful muti-nozzle 3D printer is the kernel for a successful organ 3D bioprinting with respect to the predesigned architectures (or shapes), cellular (or histological) components, and biological (or physiological) functions. The powerful muti-nozzle 3D printer can control the following processes: (1) homogeneous and/or heterogeneous distribution of multiple cell types and/or cell-laden biomaterials (including stem cells, growth factors, and other bioactive agents) in a predefined 3D construct; (2) a perfusable vascular network, nervous network and/or other special structures in the particular 3D construct; (3) multiple functionality realization with both natural and synthetic polymers for various biomedical or clinical purposes, such as multicellular accommodation or delivery, structural integrity maintenance and anti-suture anastomoses.

For each organ 3D bioprinting, the selection of appropriate polymerous “bioinks” (i.e., starting biomaterials), such as 3D printable polymers and cell types is essential to obtain desired biological functions. As stated above, the printability of the cell-laden gelatin-based hydrogels depends largely on the physical and chemical properties of the gelatin-based hydrogels (this was demonstrated in our previous studies). The solidification property of the gelatin solution at certain temperature (below 28 °C) plays a critical role in providing the 3D structural integrity as well as facilitating the cell viability [13,14,15,64,65,66,67,68,73,74,75,76,77].

### 4.5. 3D Bioprinting Process

During the organ 3D bioprinting process, a multi-nozzle 3D printer can successively layer the two-dimensional (2D) sections to produce 3D constructs with sufficient precision and accuracy. It is imperative that the polymerous “bioinks” are injected (or extruded), deposited, solidified, and layered with certain spatial resolution. Chemical crosslinking of the pertinent polymers before, during or after the 3D bioprinting processes is necessary to keep the integrity of the 3D constructs. Associated printing parameters, such as the shear stress, extrusion speed and nozzle size may have negative effects on cellular behaviors. A comprehensive understanding each of the bioprinting parameters impacting on the cellular behaviors and optimization the printing parameters are fundamental for a special organ 3D bioprinting process.

Cell density is another critical factor for the effective cell-cell, cell-environment communications and biological functionality realization. Sometimes it is necessary to let the cells undergo proliferation and/or differentiation phases before forming homogeneous or heterogeneous tissues. Especially, stem cells encapsulated in the 3D construct can be cultured for a relatively longer period to achieve an ideal density or population [13,14,15,64,65,66,67,68,73,74,75,76,77].

### 4.6. Post-Printing Organ Maturation

The term “maturation” was borrowed from botany by Prof. Wang and others in 2003. The maturation processes of different organs vary from each other depending largely on the main parenchymal tissues and the complexity of the internal/external hierarchical architectures. After 3D bioprinting, cells in the 3D construct need time to form tissues. Meanwhile, multiple tissues need time to coordinate and to be functional. Post-culture of the 3D construct may be necessary for the vascular network formation and in situ perfusion. Pulsatile bioreactor is a promising candidate for the orientational arrangement of living cells. The role of physical force for different tissue remodeling may totally differ in different organs. It may take a long period of time to coordinate the maturation frameworks of different tissues in a particular organ [13,14,15,64,65,66,67,68,73,74,75,76,77].

By taking advantage of stem cells/growth factors and multi-nozzle 3D bioprinters, heterogeneous vascularized tissues and/or organs have been first generated in Prof. Wang’s laboratory according to the specific locations in natural organs under the instructions of CAD models (Figure 5). This pioneering work is a historical mark which shows that “organ manufacturing” has entered a brand new era, when all the bottleneck problems, such as the in vitro large tissue generation, stem cell full differentiation, circular vasculature construction, branched nervous network incorporation, anti-suture anastomosis implantation, in situ nutrient supply and metabolite elimination, which have perplexed (daunted or confused) “tissue engineers” for more than two decades, have been overcome one by one by this unique group [13,14,15,64,65,66,67,68,73,74,75,76,77]. It is believed that these remarkable breakthroughs will bring huge benefit to all aspects of human health.

## 5. Gelatin-Based Hydrogels for Organ 3D Bioprinting

### 5.1. Gelatin-Based Hydrogels for 3D Bioprinting

Over the last decade, extrusion-based 3D bioprinting technologies have been emerged in various organ 3D bioprinting areas, while the gelatin-based hydorgels have received a lot of attention due to their unique physical, chemical, biological and clinical properties (Table 1) [83,84,85,86,87,88,89,90,91,92]. The gelatin-based hydrogels can be printed either as sacrificed (or fugitive) “bioinks” for channel or pore creations or solid constructs for cell survival accommodations. The extrusion-based 3D bioprinting technologies have been advanced in using the gelatin-based hydrogels to produce solid 3D structures with numerous physical, chemical, material, biological, physiological and clinical functions. Based on the data in Table 1, it can be concluded that a slight change in the ingredients of the gelatin-based hydrogels can significantly alter the final properties of the 3D printed objects.

As stated above, the melting point of the gelatin-based hydrogels is about 28 °C. This special thermally responsive characteristic of gelatin-based hydrogels is vital important for the extrusion-based organ 3D bioprinting technology in which the cell-laden gelatin-based hydrogels take different shapes at a certain temperature (such as below 10 °C).

During the extrusion-based organ 3D bioprinting processes, cells and other biomaterials (including bioactive agents) are mixed before being printed into 3D constructs with predefined internal/external topological features (Figure 3 and Figure 5). There are several factors that determine the gelatin-based hydrogels to be printed and layered during the 3D printing processes. Among all the factors, mechanical properties of the gelatin-based hydrogels determine whether the printed 3D constructs can be handled properly in vitro and/or implanted stably in vivo.

Generally, mechanical properties of the physically crosslinked gelatin-based hydrogels depend largely on the concentration of the primary gelatin solution. As a thermo-sensitive polymer, the tensile strength of the gelatin solution reduces sharply when the temperature is enhanced above 28 °C. The rigidity of the gelatin solution is not determined only by the temperature. The concentration, pH, and the presence of any additive all have effects on the value. It is the molecular structure, water content (or viscosity), together with the average molecular weight (mass) that define the value [41,42]. Nevertheless, the gelation of the gelatin-based solutions is a physical crosslinking process in which a sol-gel transformation occurs due to the conformational transition of the gelatin molecules. The mechanical properties of the physically crosslinked gelatin-based hydrogels are extremely weak. When the 3D printed constructs are put into the culture medium at room temperature, they collapse immediately due to their special thermo-responsive property.

To date, various approaches have been explored to improve the mechanical strengths and structural stability before, during or after the 3D bioprinting processes. One of the effective approaches is physical blending. For example, gelatin, blended with methacrylate (i.e., methacrylamide/gelatin, GelMA), effectively improves the viscosity of the composite (or hybrid) hydrogel. Van Den Bulcke, et al., reported that the increase of gelatin or GelMA proportion can elevate the viscosity of the hybrid alginate/gelatin or alginate/GelMA polymers and the printability of the 3D bioprinting processes [93]. Photopolymerization of GelMA with water-soluble photoinitiator and UV-light can significantly increase the mechanical strengths of the composite hydrogels and the shape fidelity of the 3D printed constructs [86]. Schuurman et al., printed chondrocyte-laden GelMA hydrogel into multiple-layer structures with a cell viability of 83% [94]. Wüst et al., created a hollow structure to facilitate cells to migrate, proliferate and differentiate [95]. The GelMA hybrid hydrogels have been employed in skin, bone, cartilage and vasculature 3D bioprinting. However, the photopolymerized GelMA and its similars or derives, such as 4-arm poly(-ethylene glycol)-tetra-acrylate (PEGTA), and gelatin ethanolamide methacrylate (GE-MA)-methacrylated hyaluronic acid (HA-MA) (GE-MA-HA-MA), are non-biodegradable superpolymers (or supermolecules) which normally have very strong hardness and limited usage in bioartificial organ 3D bioprinting areas.

Another effective approach is to chemically or biochemically (i.e., enzymatically) crosslink the polymer chains (i.e., molecular strands). The polypeptide chains of the gelatin molecules can be chemically or biochemically crosslinked to form larger macromolecules. Most of the enzymatic crosslinks (such as tyrosinase, transferase, and tyrosinase) are catalyzed at neutral pH, and benign temperature. These crosslinks maybe helpful in the future organ 3D bioprinting areas. Some chemical agents, such as aldehydes (e.g., glutaraldehyde or glyceraldehyde), genipin, polyepoxides and isocyanates, have been utilized to crosslink the gelatin-based hydrogels [96,97]. When the polymeric chains are chemically crosslinked, covalent bonds formed between the free amino groups of lysine and hydroxylysine or carboxyl groups of glutamate and aspartate in the gelatin molecules, ensuring a relative stable 3D construct. Nevertheless, some of the chemical crosslinking agents (or reagents), such as the aldehydes, are toxic to cells. Caution should be taken before cells are surrounded by the toxic crosslinkers even embedded in the hydrogels. Furthermore, one should be aware that large quantities of toxic reagents, such as the glutaraldehyde, glyceraldehyde and isocyanate molecules, may be released into the body and cause vice reactions during the in vivo degradation of the chemically crosslinked gelatin-based hydrogels.

### 5.2. Successful Gelatin-Based Organ 3D Bioprinting Technologies

Utilizing the advantages of multi-nozzle extrusion-based 3D printers and thermo-reversible properties of the gelatin-based hydrogels, Prof. Wang and others have developed a series of organ 3D bioprinting technologies that enables us to directly deposit multiple living cells, supportive materials, growth factors and/or other bioactive agents in the right position, at the right time, and with the right amount, to form 3D living organs for in vitro culture and/or in vivo implantation (Figure 3). In these approaches, the digital data of a 3D image defined in the predesigned CAD models can be translated into actual entities. Consequently, bioartificial organs possessing essential biological, biochemical and physiological functions, such as nutrient supply, protein secretion, waste discharge, anti-suture implantation, have been completed. Various perfusable vascular networks, that integrate the merits of both natural and synthetic polymers for effective cellular accommodation, tissue formation and organ maturation have been produced successfully [13,14,15,64,65,66,67,68,73,74,75,76,77].

As an alternative approach to using the toxic crosslinking agents to crosslink the gelatin molecules, several chemical crosslinkable polymers, such as alginate, chitosan, fibrinogen and hyluronan, have been added to the gelatin solutions and made the crosslinking procedures more cell friendly. The gelatin-based hydrogels, such as gelatin/alginate, gelatin/fibrin, gelatin/hyaluronan and gelatin/alginate/fibrinogen, developed by Prof. Wang in Tsinghua University have profound influence in the extrusion-based organ 3D bioprinting technologies. For example, alginate is a family of polysaccharides obtained from brown alginate which can be chemically crosslinked by divalent cations, such as calcium (Ca^2+^), strontium (Sr^2+^), and barium (Ba^2+^) ions [19]. Chitosan, derived from the deacetylation of chitin, is a positively charged amino polysaccharide (poly-1, 4 d-glucoamine), which can form a polyionic complex with the negatively charged gelatin-based hydrogels with ionically interacts and can be crosslinked by sodium tripolyphosphate (TPP) [18]. Hyaluronic acid or hyaluronan (HA) is another major constituent of the ECMs in the human tissues, such as the skin, lens, and cartilages. It can bind other large glycosaminoglycans (GAGs) and proteoglycans through specific HA-protein interactions and can promote the migration and growth of cells. HA can be enzymatically crosslinked [21]. Fibrinogen is a monomeric protein with a molecular weight of about 340,000 Daltons, generated when the fibrinogen peptide A and B are resected by thrombin in the coagulation process, which have been widely used as haemostatic wound dressing materials in the forms of sponge, film, powder or sheet [20,27,28]. These natural polymers have been successfully applied in the extrusion-based 3D bioprinting technologies to produce biocompatible, biodegradable, and biostable 3D constructs with predefined architectures [13,14,15,64,65,66,67,68,73,74,75,76,77]. The predefined architectures act as temporary organ generation templates for guiding the cellular activities, such as migration, proliferation, self-organization and differentiation (Figure 5).

### 5.3. Challenges for Complex Organ 3D Bioprinting with a Whole Spectrum of Physiological Functions

Organ 3D bioprinting is a comprehensive process comprised of many physical and chemical changes of the starting materials. It is also a developing and crossing multidiscipline that involves many sciences and technologies, such as materials, biology, physics, chemistry, mechanics, computer and medicine. Like the gelatin-based hydrogels which have been used in various 3D bioprinting technologies, each organ can be printed in some special ways with regard to the 3D printer types, “bioink” compositions, and clinical applications. To print a bioartificial organ with a whole spectrum of physiological functions, some challenges still exist. These challenges can be analyzed from the following three aspects.

Firstly, from the 3D printer aspect, two of the major hurdles are the out of date soft- and hardware. At present, most of the existing 3D bioprinters can only print one or two “bioinks” (i.e., starting materials) at one time with limited structural, material, and biological functions. Especially the most powerful extrusion-based 3D printers equipped with only one or two nozzles, which have greatly limited their usage in multiple material selection, hierarchical structure construction and critical function implementation. It is realized that different nozzles may have different usages in the extrusion-based 3D bioprinting. Meanwhile, different 3D bioprinting technologies may have different merits in complex organ manufacturing areas. To overcome these hurdles, combined multi-nozzle 3D printers with updated soft- and hardware are expected to provide new feasible ways for highly complicated organ 3D bioprinting. These combined multi-nozzle 3D printers should be endowed with additional capabilities to fulfill extra tasks in a target organ 3D bioprinting [79,98].

Secondly, from the “bioink” selection aspect, two of the major drawbacks until now are the insufficient tissue/organ growth abilities (with enough cell density and population) and the unmatched mechanical properties of the vascular networks. This includes the polymer systems and cell resources. Only a few existing polymers, like the PU and gelatin-based hydrogels, can meet all the basic requirements for a successful organ 3D bioprinting with excellent biocompatibilities, exclusive processibilities, desired mechanical properties and tunable biodegradabilities. Many more new polymers or polymer combinations need to be exploited. Using sacrificial polymers is an intelligent strategy to provide extra structures in a target organ. Polymers, whether biodegradable or non-biodegradable, can be used for artificial organ manufacturing. Non-biodegradable polymers have limited usage in bioartificial organ manufacturing areas. It is also expected that one type of stem cells derived from the patient can solve all the cell source problems. When stem cells are used in the “bioinks”, the carcinogenic and teratogenic potentials of homologous and heterologous stem cells should be properly controlled. Those growth factors, whether they are mixed with the stem cells before 3D bioprinting or applied to the post-printing culture medium, should be optimized for a typical organ 3D bioprinting technology [81,99].

Thirdly, from the physiological function aspect, the major challenge to date is still the establishment of the perfusable hierarchical vascular networks. A perfusable vascular network is vitally important to obtain bioartificial organs that can be implanted in vivo. A fully confluent endothelial layer on the inner surface of the vascular lumen is a prerequisite for the antithrombotic properties. A further issue is the adaption of the vascular network to the host to allow in situ perfusion of the 3D construct and assure the survival of the cellular constituents within. Caution should be taken that the successful in vitro models cannot guarantee the in vivo clinical applications. Difficult as it is, the hybrid PU/gelatin-based cell-laden hydrogels or the synthetic polymer, such as PU and PLGA, encapsulated gelatin-based cell-laden hydrogels have been demonstrated to have a promising future [100,101].

## 6. Conclusions and Perspectives

Generally, there are four steps in a typical organ 3D bioprinting process: blueprint predesigning, 3D printer and “bioink” preparation, processing and post-printing maturation. Multi-nozzle extrusion-based organ 3D bioprinting technologies have demonstrated many distinguished advantages (such as automated, sophisticated and integrated) in “organ manufacturing” areas. Gelatin-based hydrogels, such as the gelatin/fibrinogen, gelatin/alginate/fibrinogen, and gelatin/alginate/fibrinogen/hyaluronan, with remarkable physical, chemical, biological and medical features, have played a vitally important role in each of the successful extrusion-based organ 3D bioprinting technologies. A combined multi-nozzle 3D printer can be used to manufacture highly complicated organs with a variety of desired morphological structures, cellular components, and physiological functions, such as vascular and nervous networks. A biologically mimicking CAD model derived from patient or experience can be used to guide the customized or high throughput organ 3D bioprinting processes.

In the future, bioartificial human organ manufacturing, by using the combined multi-nozzle extrusion-based 3D bioprinting technology will be very popular in many laboratories throughout the world. More sophisticated techniques, including µCT, MRI and X-ray techniques can be integrated for the clinical datum collection, analysis, usage and storage. Modern imaging techniques, such as caged fluorophores and quantum dots will allow scientists to visualize the morphological changes of cells, tissues and organs occurring in a predefined 3D construct in real time. Much more structural, material, pathological and clinical features in each individual organ, such as the nephric tubules in the kidney, alveolus pulmonis in the lung and lactiferous ducts in the breast, will be incorporated to ensure the mass cells are functional. Computers will make the datum storage, manufacture process and architecture predesign much easier. Bioartificial organs automatically produced through the 3D bioprinting technologies with much more hierarchical structures, particular cell types, bioactive ingredients and physiological functions will play a pivotal role in regenerative medicine and other pertinent biomedical fields, such as defect/failure organ restoration, high-throughput drug screening and metabolic syndrome model establishment. Numerous patients will benefit significantly from these prominent organ 3D bioprinting technologies, theories and principles. The average longevity of human beings will definitely be prolonged miraculously through the defect/failure organ restoration applications, effective drug screening protocols and disease metabolism controlling templates.

## Figures and Tables

**Table 1:** Resume of gelatin-based hydrogels for organ 3D printing.

3D Bioprinting Technology	“Bioink” Formulation	Crosslinking Method	Application	Morphology	Ref.
One nozzle extrusion-based 3D bioprinting developed in Tsinghua University Prof. Wang’s laboratory	Gelatin/hepatocyte	2.5% glutaraldehyde solution	Large scale-up hepatic tissues	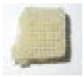	[17]
	Gelatin/chitosan/hepatocyte	3% sodium tripolyphosphate (TPP) solution	Large scale-up liver tissues	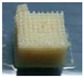	[18]
	Gelatin/alginate/hepatocyte; Gelatin/alginate/chondrocyte	10% calcium chloride (CaCl_2_ or Ca^2+^ ion) solution	Large scale-up hepatic and cartilage tissues	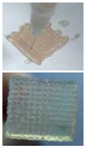	[19]
	Gelatin/fibrinogen/hepatocyte; gelatin/fibrinogen/human neonatal dermal fibroblast and mesenchymal stem cell	Thrombin induced polymerization	Large scale-up hepatic tissues; vascular channels	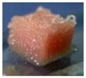	[20]
	Gelatin/hyluronan	2% glutaraldehyde solution	Brain defect repair; cell attachment	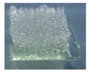	[21]
	Gelatin/alginate/adipose-derived stem cell (ADSC)	5% CaCl_2_ solution	Vascular networks	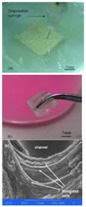	[22]
	Gelatin/alginate/ADSC-laden microcapsule	Double crosslinking (100 mM/L CaCl_2_ for ADSC-laden microcapsule; 5% CaCl_2_ for microcapsule containing grid structure)	Vascularized tissues and organs	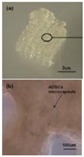	[23]
Two-nozzle extrusion-based 3D printing developed in Tsinghua University Prof. Wang’s laboratory	Gelatin/alginate/fibrinogen/ADSC-gelatin/alginate/fibrinogen/hepatocyte; Gelatin/alginate/fibrinogen/endothelial cell-gelatin/alginate/fibrinogen/muscle smooth cell	Double crosslinking with CaCl_2_ and thrombin solutions	Vascularized liver and adipose tissues	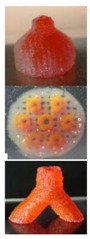	[25,26]
	Gelatin/alginate/fibrinogen/ADSC-gelatin/alginate/fibrinogen/pancreatic islet	Double crosslinking with CaCl_2_ and thrombin solutions	Vascularized adipose, hepatic and cardiac tissues	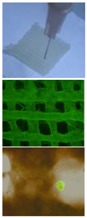	[27,28]
	Gelatin/alginate/fibrinogen/HepG2; gelatin/alginate/fibrinogen/hepatocyte or gelatin/alginate/fibrinogen/hepatocyte/ADSC	Double crosslinking with CaCl_2_ and thrombin solutions	In vitro liver tumor model establishment and anti-cancer drug screening	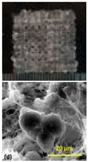	[38,39]
Two-nozzle low-temperature extrusion-based 3D printing developed in Tsinghua University Prof. Wang’s laboratory	Gelatin/lysine and polyurethane (PU) either being printed overlapped or alternated	Freeze drying (or lyophilization) for solvent sublimation (or structural stabilization) and 0.25% glutaraldehyde for gelatin/lysine crosslinking	Bioartificial organ manufacturing with expected (or controlled) mechanical properties and interconnected channels	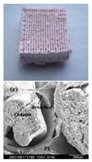	[68]
	PU-ADSC-PU; PU- ADSC/gelatin/alginate/fibrinogen hydrogel	Double crosslinking with CaCl_2_ and thrombin solutions	Tubular and sandwich-like PU-ADSC/hydrogel-PU; implantable branched vascular templates	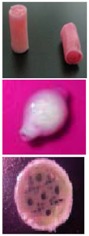	[32,33]
Dual-syringe Fab@Home printing device	Gelatin ethanolamide methacrylate (GE-MA)-methacrylated hyaluronic acid (HA-MA) (GE-MA-HA-MA)/HepG2 C3A, NIH 3T3, or Int-407 cell	Ultraviolet (UV) light (365 nm, 180 mW/cm^2^) photocrosslinking	Tubular hydrogel structures for cell attachment	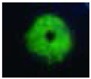	[83]
One-nozzle extrusion-based 3D bioprinting	Gelatin/alginate/myoblast	CaCl_2_ solution	Muscles	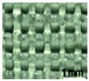	[84]
Fab@HomeTM (one-syringe extrusion-based 3D printing)	Gelatin/alginate/smooth muscle cell (SMC)/aortic valve leaflet interstitial cell (VIC)	10% CaCl_2_ solution	Aortic valve conduits	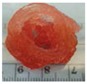	[85]
NovoGen MMXTM, Organovo (one-nozzle extrusion-based 3D printing)	Gelatin-methacrylate or methacrylated gelatin (GelMA)	Photopolymerization by exposing GelMA precursors to UV light (360–480 nm) at 850 mW (Lumen Dynamics) using 0.5% (*w/v*) 2-hydroxy-1(4-(hydroxyethox) phenyl)-2-methyl-1-propanone photo initiator	Branched vascular templates; vascularized osteogenic tissue	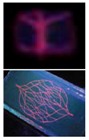	[86,87]
An inkjet-based 3D bioprinter	Gelatin and human umbilical vein endothelial cell (HUVEC) mixture act as a fugitive template	None	A hollow for HUVEC attachment	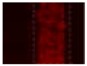	[88]
One-syringe extrusion-based 3D printing	Nanosilicate/GelMA	UV light (320–500 nm) for 60 s at an intensity of 6.9 mW/cm^2^	Electrical conductive	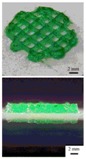	[89]
EnvisionTEC 3D-Bioplotter^®^	Polyethylene glycol (PEG)/gelatin-PEG/fibrinogen	Gelatin scaffolds were cross-linked with 15 mM EDC and 6 mM NHS, fibrinogen-containing samples were treated post-printing with 10 U/mL thrombin in 40 mM CaCl_2_ for ~30 min	Grid structures for cell seeding	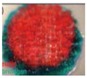	[90]
Combined four-nozzle 3D bioprinting developed in Tsinghua University Prof. Wang’s laboratory	Poly(lactic acid-*co*-glycolic acid) (PLGA)-gelatin/alginate/fibrinogen/ADSC-gelatin/chitosan/hepatocyte-gelatin/hyaluronate/Schwann cell	Double crosslinking with CaCl_2_ and thrombin solutions	Implantable vascularized and innervated hepatic tissues	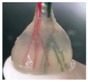	[34]
Two-syringe Fab@Home printing device	A sacrificed multi-layer (six layers) lattice gelatin/glucose construct, each layer covered with a layer of hepatocyte containing alginate hydrogel	Crosslinking with CaCl_2_ solution	Large scale-up tissues	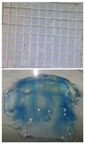	[91]
Multiple cartridge extrusion-based 3D printer	Polycaprolactone (PCL)-gelatin/fibrinogen/hyaluronic acid/glycerol	Thrombin induced fibrinogen polymerization	Bone, cartilage and skeletal muscle tissues	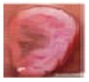	[35]
A multilayered coaxial extrusion system	A specially designed cell-responsive bioink consisting of GelMA, alginate, and 4-arm poly(-ethylene glycol)-tetra-acrylate (PEGTA)	First ionically crosslinked by calcium ions (Ca^2+^ ion) followed by covalent photocrosslinking of GelMA and PEGTA	Perfusable vasculature	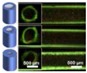	[92]
